# “It's unrealistic”: experiences of Swedish elite female ice hockey players considering parenthood

**DOI:** 10.3389/fspor.2025.1548261

**Published:** 2025-02-27

**Authors:** M. Bergström, R. Høigaard, N. P. Aspvik, S. A. Sæther

**Affiliations:** ^1^ Department of Sociology and Political Science, Norwegian University of Science and Technology, Trondheim, Norway; ^2^ Department of Sport Science and Physical Education, University of Agder, Kristiansand, Norway

**Keywords:** performance, career transitions, dual role, work-life balance, women's ice hockey

## Abstract

**Introduction:**

The number of elite female athletes combining athletic careers with mothering has increased during the last decades. Many mother-athletes return to an equal or an even better level of performance postpartum. Despite numerous success stories across a variety of sports, there are currently no mother-athletes in Swedish elite ice hockey. Therefore, the aim of this study was to explore how Swedish elite female ice hockey players perceive challenges associated with initiating and maintaining the mother-athlete role.

**Methods:**

Qualitative data were collected through semi-structured interviews with 7 elite female ice hockey players (5 prepregnancy and 2 mothers/former athletes) and analyzed using thematic analysis.

**Results:**

Thematic analysis revealed three main challenges facing elite female ice hockey players: (a) *Making ends meet*, (b) *A leap in the dark* and (c) *You can't be what you can't see*. Due to these challenges, the participants perceived combining elite ice hockey and mothering as unrealistic. Consequently, they felt forced to either retire from elite sports early or postpone mothering to post career.

**Discussion:**

Continued work with improving the financial support and developing clear maternity policies is essential to prevent early dropouts before elite female ice hockey players have reached peak performance. Enabling more players to prolong their athletic careers during pregnancy and postpartum will likely produce more positive mother-athlete role models. Further, keeping more players in the sport for a longer period could help women's ice hockey to develop to its highest optimal quality (e.g., level and competition).

## 1 Introduction

The record high female participation in the Paris Olympic Games (e.g., 50:50 representation) marks a new era for the sports movement in the work towards gender equality (1, 2). Even if there is big financial gap between male and female athletes (e.g., soccer), the latest Summer Games exemplifies the growing trend of female participation in elite sports (2). The development has made more elite female athletes to continue to compete throughout their 20s and 30s [e.g., (3, 4)]. Consequently, for many athletes, their most competitive years will coincide with the biological window of opportunity for having children (4–6). In the last decades, the number of mother-athletes has increased across various sports (7, 8). Several studies (3, 5, 9–11) show examples of athletes who have returned to an equal or an even better level of performance after a pregnancy. Mother-athletes have also received an increased media coverage, which have shed light on perceived challenges and as well as benefits associated with mothering (12–14).

Previous research show that mothering can have positive mental impacts such as increased well-being and work-life balance among elite female athletes (5, 15). Further, mothering can reduce performance pressure (9, 16) and spark new training motivation (11), which can prolong the athletic career by several years (5). Nevertheless, there are still multiple challenges facing female athletes and considering motherhood (5). For example, previous research has reported challenges such as lack of maternity rights from sport federations (5, 6, 17), vague contract conditions (e.g., worry about deselection) (18, 19) and financial instability (e.g., loss of sponsors) (3, 8, 20). Furthermore, many athletes perceive a lack of specific training recommendations during pregnancy and postpartum (5, 8), limited coach support (20), and negative cultural notions about the mother-athlete role (e.g., assumptions that pregnancy signifies the termination of the athletic career) (6, 19, 21). Additionally, when returning to elite sports postpartum, many athletes experience role tensions caused by the dual commitments (e.g., mother vs. athlete) (5, 22). Hence, becoming a mother-athletes can be a double-edged sword.

### 1.2 Women's ice hockey

Ice hockey is one of the most popular winter sports in the world with over 1.5 million players (23). Even if male athletes still dominate the sport, the number of female players as well as the commercial interest in women's ice hockey has increased dramatically after the sport joined the Olympic program in 1998 (24). Just between the years 2007 and 2018, the number of registered female players worldwide grew from 153,665 to 205,674 (25). In the last decade, women's ice hockey has also taken its first step towards professionalization which can be seen in the Swedish Women's Hockey League (SDHL) and the newly launched Professional Women's Hockey League (PWHL) in North America (26, 27). Despite the ongoing transformation, women's ice hockey is still scarcely explored in the research literature.

Even if there are numerous of examples of female athletes across various sports (e.g., football, swimming, cross-country skiing, track and field, and distance running) who successfully combine elite sports with mothering (5, 7, 8), there are currently no mother-athletes in any of the 10 SDHL teams in Sweden (28). This can be compared with the corresponding men's league, the SHL, where over 40% of the players have children (29) or the national average (12%) among Swedish elite female athletes (30). Given that the Scandinavian societies traditionally are known for emphasizing social and gender equality, providing generous parental leave opportunities and public care services for children and the elderly, and boasting a relatively high political representation of women (31), the lack of mother-athletes in the SDHL could be seen as rather surprising. Therefore, the aim of this study was to explore how Swedish elite female ice hockey players perceive challenges associated with initiating and maintaining the mother-athlete role.

**Research question:** *How do Swedish elite female ice hockey players perceive challenges associated with initiating and maintaining the mother-athlete role?*

## 2 Methods

### 2.1 Participants

Two categories of elite female ice hockey players were recruited to this study; (a) prepregnancy athletes with a wish to become a mother at some point in life (requirement to participate in the study) and (b) current mothers who had discontinued their athletic careers in conjunction with having children. Initially, the authors also wanted to include current mother-athletes in the study. However, the authors had to adjust to the fact that there were no mothers in the league. “Elite” in the present study was defined as Tier 4 athletes (32) playing in the highest division in Sweden (e.g., the SDHL). To identify prospective athletes with characteristics matching the inclusion criteria, the researchers contacted the general managers or other staff members from six different SDHL clubs. From the six club who were contacted, only one replied to the researchers’ request. Therefore, to find more participants, the first author (M.B.) also reached out directly to players through personal contacts within the ice hockey community. The prospective athletes were contacted through email. In total 7 athletes (5 prepregnancy athletes and 2 mothers, M = 26.57 y; SD = 3.9) from three different SDHL clubs accepted and fulfilled the requirements (see Table 1). Before the participants provided their consent to participate, they confirmed that they matched one of the two categories for inclusion (e.g., being a mother or wanting to have children at some point in life). Several of the players had experience from representing the national team and playing international tournaments. However, this was not a part of the inclusion criteria.

**Table 1:** A descriptive overview of the participants.

Pseudonym	Age	Category	Current occupation(s)
Sarah	20–24	Prepregnancy	Student-athlete
Johanna	20–24	Prepregnancy	Full-time athlete
Emelie	20–24	Prepregnancy	Working-athlete
Wilma	25–30	Prepregnancy	Full-time athlete
Amanda	25–30	Prepregnancy	Working-athlete
Maja	31–35	Mother	Former athlete
Vera	31–35	Mother	Former athlete

### 2.2 Data collection and analysis

Data was collected through semi-structured interviews lasting 19–35 min (M = 27.09; SD = 11.4). Notably, the recorded interview time did neither include initial “getting to know you” questions nor the closure of the interviews. This would have added approximately 15 min to each interview. The participants were based in various locations in Sweden (e.g., on training camps). Therefore, the interviews were conducted using online video conference software (Zoom Video Communications, Inc.). The authors also considered this as more time efficient and flexible for the researchers and the participants (33). To collect data with “information power” (34), the interview guide included topics relating to the informant's athletic background, family life, economic situation, and preconceptions, experiences, and perceived challenges related to the mother-athlete role. Some of the questions from the interview guide were: (a) In what way did/do you expect having a baby affect/will affect your athletic career? (b) What do/did you find difficult with being/becoming a mother-athlete? (c) What would you need to make the mother-athlete role work? The interviews were conducted by the first author (M.B.).

The collected data was recorded and transcribed before analyzing it according to the six steps of thematic analysis (35, 36): (a) familiarizing yourself with the data, (b) generating initial codes, (c) searching for themes, (d) reviewing themes, (e) defining and naming themes and (f) producing the report. Thematic analysis provides a systematic data management which facilitates a well-structured final report to be produced (35). After transcribing the interviews, the first author (M.B.) read the transcripts to get a general sense of the material (a). In the next step, some interesting features (e.g., the everyday life, financial situation, dual career challenges, perceived uncertainty and notions on mothering and elite sports) were bunched into different categories (b) and labelled (c). In step 4 (d), the preliminary categories were reviewed after discussing them from different research perspectives and implications with the co-authors. Next, the themes were refined and labelled into three main themes (e): (1) *Making ends meet*, (2) *A leap in the dark*, and (3) *You can't be what you can't see.* Finally, quotes that reflected the themes and the study aim were selected and presented in the report (f). To ensure peer validity, all authors discussed various perspectives and interpretations of the themes.

### 2.3 Rigor

The present study used a descriptive approach which is a useful method for researchers wanting to know the “*who?*”, “*what?*”, and “*where?*” of specific events. Compared to other approaches (e.g., phenomonologic, ethnographic or narrative), qualitative descriptive studies are especially useful for researchers who desire a straightforward description of the explored phenomena by staying close to “the surface of words and events” (37). Qualitative descriptive studies may rightfully be described as the least theoretical of the spectrum of qualitative approaches. Despite being criticized for being trivial or superficial, other argue that conveying participantś perceptions of a phenomenon in a cohesive and useful way is neither trivial nor easy at all. Rather, a good descriptive summary of the explored phenomenon does not only give answers to the research question but can also be “most relevant to the audience for whom it was written” (37). Additionally, being less encumbered by pre-existing theoretical and philosophical commitments may sometimes be an asset in the research process. Hence, the present study should be seen as a qualitative description of challenges associated with initiating and maintaining the mother-athlete role among elite female ice hockey players.

To ensure high-quality qualitative research, the authors conducted the study based on the guidelines advocated by Tracy (38). The authors ensure that the eight criteria; (1) *worthy topic,* (2) *rich rigor,* (3) *sincerity,* (4) *credibility,* (5) *resonance,* (6) *significant contribution,* (7) *ethics,* and (8) *meaningful coherence* were adhered to. For example, before collecting the data, the authors discussed the focus of the study (worthy topic), the selection of context and the interview guide topics (rich rigor). Further, during the data analysis, the authors continuously sought to discuss possible interpretations of the collected data (credibility) and engaged in critical self-reflection in which they tried to be aware of potential biases and predispositions (sincerity) (39). Finally, even if the recruitment of participants proved to be more challenging than first expected, we argue that the collected data has high “information power” due to the specific experiences of the participants (34).

### 2.4 Philosophical position

The present study is positioned within a social interactionist ontology (39). Further, the study adopts a constructionist approach, which suggests that multiple realities exist, and that social reality is a subjective experience (40). For example, by letting the participants construct their reality within their contexts, we hope to understand how they negotiated their priorities in relation to elite sport and mothering, as well as how contextual factors on the meso- (e.g., club and teammates) and macro levels (e.g., sport federation, national culture, sport culture) interacted with these perceptions. Hence, the authors never attempted to find an absolute “truth”.

The authors have many years of experience of conducting qualitative research on talent development environment and female specific challenges in elite sport. Further, the authors have own experience as from being athletes and/or coaches in a variety of sports (e.g., soccer, ice hockey, handball, cycling and cross-country skiing). Additionally, three of the authors have children, which provided them with an understanding of some of the challenges that parenthood adds to the “life puzzle”. This demonstrates the authors’ in-depth understanding of the subject explored in the present study, which qualified them to collect and interpret the data for this study.

### 2.5 Ethical statement

Before the interviews were conducted, all participants received an information letter containing all information about the study objective, interview topics, procedures and confidentiality. All participants were also informed that participating in the study was voluntary and that they could withdraw at any time during the process until the findings were published without an excuse. Before the data was analyzed, all participants received their interview transcript and were given an opportunity to add, remove or correct information. To avoid that the participants could be identified through the selected citations presented in the findings, they were given pseudonyms which only the researchers had access to. Further, any specific information that potentially could reveal the identity of the informant (e.g., age, teams, names of children, partners, teammates, coaches or specific athletic achievements) was rewritten or removed. The study was carried out according to the Declaration of Helsinki and approved by the Norwegian Agency for Shared Services in Education and Research (Ref.nr. 118248).

## 3 Results

Thematic analyses generated three main mother athlete-specific challenges facing elite female ice hockey players when considering mothering in terms of: (a) *Making ends meet*, (b) *A leap in the dark* and (c) *You can't be what you can't see*.

### 3.1 Making ends meet

All the participants had signed professional contracts with their clubs which gave them some financial support. However, only two of the seven participants considered that they could be fulltime athletes and did not work extra alongside the athletic career. Even so, the two full-time athletes (Johanna and Wilma), felt that the money from the athletic career wouldn't be enough to provide for a family. Instead, they described that their current salaries just covered the monthly costs for rent and food and that they were considering getting an additional non-sport related job or to become student-athletes. For the other participants, the everyday life was described as a somehow manageable haste. This was described by Amanda:

I get up at 5:45am, I go to work, then I always have lunch at the arena and stay there and work until practice starts. If we have double sessions I go to practice in the morning and stay there and work after practice to lunch time, eat there and continue working until the next practice starts. Sometimes I need to catch up with some work in the evening and then I continue working when I get home, so there is little else than hockey and work in my life right now! (laughing)

Even if Amanda perceived her everyday life as busy, the fact that she could work remotely seemed to facilitate the dual career. For most of the participants, relying solely on the income from ice hockey was not considered enough to make ends meet at the end of the month. For example, the two mothers in the sample both played professionally for 10 years but always combined their athletic career with fulltime work or fulltime studies. Even if they had professional contract, their income earned from sports had never been more than 3,000 SEK/month (∼270 USD). This was far from enough to be able to pay the rent or to put away savings for the future. Additionally, Maja and Vera both described that salary earned from the athletic career had neither qualified them to get financial support from the state during a potential maternity leave nor entitled them to future pension. As they had reached the age of 30 and started to consider having children, Maja and Vera had found themselves in a road crossing, as described by Maja:

I do think it was worth it with all the experiences, memories and travels I've done with the team. It's a lot of laughter and I wouldn't want to be without it but I do understand why most of the players end their career before they turn 30. It was the same for me… it was the financial challenges that were determinant. If I would have had a normal salary […] then I would have considered to play until I was 35 […] but no… It's like you reach a limit when you must start thinking about other stuff [having kids and buying a house]

Even if Maja expressed her passion for ice hockey and felt that she probably could have continued for another 5 years, the economic reality made her retire from sport early at age 28. Further, to go from an already busy dual career (e.g., work-sport) to an even more busy triple career (e.g., work-sport-family) with all the additional traveling to away games across the country was perceived by her as unrealistic. All participants perceived the economic aspects as the main challenge for continuing the athletic career as a mother-athlete. Similarly to Maja and Vera, all the prepregnancy athletes expressed that they would probably have to retire from sport earlier than they wanted, as expressed by Johanna:

As I see it now, when you want to start a family, you might not be able to play hockey for as long as you would have wanted because of the money. You might have to get an extra job to earn some more [money] and then you won't have the time to be a full-time athlete, be away, work and have a family and children…

Similarly, Sarah explained that it just wasn't about having an income to be able to provide for a child once it was born, but also to have enough time to build up an economic buffer before even getting pregnant. This, she believed, was not possible if she would still play elite level ice hockey. Hence, because of the economic concerns and the perceived incongruity of balancing the athletic career with mothering and additional work, mothering was described as career ending event.

### 3.2 A leap in the dark

None of the participants knew how their clubs and coaches would react if they became pregnant. Unlike injuries or doping issues, pregnancy was not mentioned in their contracts or a topic that was discussed proactively in the club. All participants assumed that their contracts would be broken if they got pregnant, as expressed by Sarah:

Right now, there are no safety nets if you would get pregnant. You might have a two-year contract, but no more. Let's say you got one year left, why would they [the club] want to extend it? There is nothing about maternity leave or that kind of stuff [in the contract]. You can't fulfill your part of the contract when you're pregnant…

Maja had become pregnant in the middle of the season. Although she was met by positive reactions from her coaches and teammates, she had not even considered returning to sport after her maternity leave as an option:

Everyone was happy for me. I don't think anyone thought that it was unexpected that I retired from sport. I remember that my coach called me and asked: “Are you ending your career?” and I said: “Yes, I'm done”. Right there, I didn't even think about what it might have been to continue playing… like how it would work with the logistics and everything…

Her experiences illuminates that the club lacked clear policies on maternity provisions and support during and after pregnancy, but also that she assumed that returning to sport was not even an option. Therefore, Maja did not seem to bother to ask the club about maternity support available during and after the pregnancy. This is similar to Vera who described that she had gotten mixed signals from her club and coaches:

We usually had 1 + 1 year [contracts] that were extended every season. We always had a meeting and one year, I almost didn't get a renewal because they [the club] thought that I was going to have kids […] but one week later they called me and said “oh, we want you anyway!”. Like a joke, a former coach could tell me like: “aren't you going to get pregnant?” or “aren't you going to have kids? Then it's OK to end the contract” but it was still a joke. I don't know what would have happened if I would have become pregnant during the season and still wanted to continue my career. I have no idea…

On the one hand, her coach seemed to encourage her to have a baby, but at the same time, even if it was told as a joke, the consequences were made clear if she broke the contract. This seemed to have made Vera even more uncertain of which kind of support which would be available from the club. Instead, Vera chose to retire from sport before she got pregnant at age 28. Vera simply assumed that pregnancy would mean breaking the contract and that there was no support (e.g., financial or training specific) or social security (e.g., maternity leave) available. This assumption was shared by all the participants. Instead, mothering was seen as choosing between two lives: ice hockey or family. The participants perceived that just bringing up the topic to the club or coaches was perceived as a potential risk of getting deselected. Amanda, one of the prepregnancy players, expressed that she ideally wanted to be able to combine her athletic career with having children in the future. Even so, she seemed to accept that the two lives would be incompatible partly because she believed that there would not be any club support available:

I mean, the dream would be to have kids but still be able to continue doing what you love [playing hockey] but it's difficult, so I feel like you must decide at some point, like: “Now Ím done” and start a new life. My partner and I have discussed it but right now my hockey career stands in the way [for starting a family]

Hence, the uncertain contract conditions and lack of communication about pregnancy and maternity leave provisions made the participants postpone having family or retiring from sport early.

### 3.3 You can't be what you can't see

A third factor that seemed to affect the participants’ attitudes on initiating and maintaining the role as a mother-athlete in a more skeptical direction was the lack of successful mother-athletes in the league. Instead, the participants described notions such as that elite and sport mothering were incompatible. These notions seemed to be based on experiences of a few former teammates. The participants described mother-athletes as a rare phenomenon that usually did not last very long, as expressed by Sarah:

There are some people I've played with who had children. One of them retired the same season that she got pregnant but returned the year after and tried to play. She tried like one season, but it didn't work well so she quitted. There was another one who tried to combine it but not long after she also quitted. Recently, there was one who got pregnant, but she announced that she wouldn't continue playing…

Wilma expressed concerns about how having a baby might affect her athletic goals. Wilma believed that she still had her best years as an athlete ahead of her and dreamed of representing her country at the Olympics and World Championships. Being in this stage of her athletic career made her less willing to “jeopardize” her performance level:

There is a lot of focusing on yourself during the elite years. You need to get enough sleep, do your training sessions, eat, nutrition and everything and then you suddenly would have to change that focus to a baby or adapt to your partner […] I believe children are time demanding and that's the life of an elite athlete too, so I believe it would be difficult to have time for both and still do it well.

To Wilma, risking her career goals by adding the stress of a child was one of her arguments for postponing family life. The notion seemed to be based on the absence of successful role models in the league. Vera described a former teammate who seemed to have managed the combination:

We had a goalie, and she used to take her baby with her, but I always though like: “how does she do that!?” because it's tough to take care of a baby, rest, prepare for the games, food… It really looked tough! When I watched her, I felt like: “no, I don't want that life” I've never tried [to be a mother-athlete] and can't really say anything about it… No, I wouldn't have had the energy. I'm not lazy, but it would be tough to take the baby with you.

Similarly to Wilma, Vera had also been skeptical towards the potential decline in performance that having a baby would mean to her. To Vera, the lack of (perceived by her) inspiring role models (e.g., successful mother-athletes in elite ice hockey) seemed to keep her from even trying to initiate the mother-athlete role. Further, all the participants seemed believe in the notion that if you had a baby, you could not be serious with your athletic career anymore, as expressed by Amanda:

When they are infants and really need their mother its challenging. I know that the teammate I use to play with breastfed her baby between the periods. I mean like, how focused can you be on the game if you're like: “the baby has to eat”? I mean, I don't know how it would have worked out even if I was a fulltime professional…

As Amanda mentioned, she doubted that it would be possible to be a mother-athlete even if she hypothetically would be able to live from her sport career financially because of the competing demands (e.g., mother vs. athlete). By contrast, all participants believed that they, with the right amount of training, would be able to return to a comparable performance level and physique after childbirth.

## 4 Discussion

The aim of this study was to explore how Swedish elite female ice hockey players perceive challenges associated with initiating and maintaining the mother-athlete role. The findings reflect the underlying thought processes that influence elite female ice hockey players' choices about postponing mothering or discontinuing the athletic career rather than initiating and maintaining the mother-athlete role. Consistent with previous research (5, 6, 19, 20) our findings revealed a range of challenges.

### 4.1 Money, money, money

One of the main findings of the present study was that the financial challenges perceived by the players. This aligns with research conducted on North American professional female hockey players (41) as well as other individual sports in Scandinavia (e.g., cross-county skiing) (5, 42). Similarly to the SDHL, it is common for players in the North American PWHL to earn or supplement their income from non-hockey related employment (41). Hence, being a dual career athlete (e.g., work and sport career) is still the reality of most professional female hockey players (43). Similar findings have also been reported from European women's elite football (44). A recent report from the Swedish labor union *Unionen* (29) showed that the average monthly salary for professional female ice hockey players is around 5,500 SEK (∼500 USD) in the SDHL. The number can be compared with the average monthly salary for professional male players in the corresponding SHL, which is around 120,000 SEK (∼11,000 USD) (29) as well as the average salaries among men (42,000 SEK) and women (37,800 SEK) in the general population (45). Hence, most professional female hockey players in the SDHL must make a substantial commitment to both their athletic careers as well as their non-athletic careers. Taking this into account, it is understandable that the players' perceived that adding the additional demands of mothering to an already full plate as unrealistic (e.g., going from dual to a triple career).

As our findings illuminate, the necessity of supplementing the income from non-sport related work likely maintained the notion that mothering is a career ending event. Wilhelmsen's (44) showed that many female elite-level football players end their athletic careers in their twenties because of the heavy workload of having dual or triple careers (e.g., elite-level sports, studies and/or work). Early athletic career termination *per se* is a challenge for the professionalization process of women's sport since many elite female athletes retire before they are fully developed in their sport (e.g., experience, competence and performance). Hence, this can hinder women's elite sports to develop to the highest optimal quality (44).

Previous research illuminates that most athletes can return to an equal or an even better level of performance after a pregnancy (3, 5, 9–11). However, in our study, all the players expressed that they either had or probably would retire from professional sport early to be able to start a family mainly due to the financial instability. It can only be speculated what consequences this has for the quality and competition of the SHDL and for women's ice hockey internationally. For example, if the most experienced players will continue to retire early, this will likely affect the quality of the game and thereby the sport's market value. If the level of the league is perceived to be too low by sport federations, clubs, sponsors and spectators, this could potentially affect their willingness to make necessary investments to change the current situation (e.g., salary raise). Rather this will create a vicious circle with poorer performances due to the players’ demanding everyday life, early career terminations, league instability, lack of legitimacy for women's ice hockey, low media interest and limited resources (43). Further, for nations with relatively low numbers of female players (e.g., Sweden, Finland and Norway), keeping as many players in the sport for as long as possible may be essential to be able to compete internationally against the top ranked countries Canada and USA (23). Here, continued work with creating sustainable working conditions for female players (e.g., financial support) is crucial for continued development of the sport (41, 43, 44). Even if there still is a big financial gap between professional male and female hockey players, the situation has started to improve for female players, which the PWHL-player Daryl Wattśs contract for the 2023/2024 season is an example of (46). Further, in Sweden, the SDHL has set the ambition to enable 25% of the female player to live from their athletic career in 2025 (27) compared to the present 7% (29). Therefore, future studies could follow the development in the coming decades.

### 4.2 Don't shut me down

Similarly to Bergström et al. (5), the players in the present study could not predict the exact consequences for their athletic careers when considering or initiating the mother-athlete role. They were uncertain about how their current contracts would be affected (e.g., risk for deselection). This aligns with previous research of both team and individual sports (6, 18, 19). Further, consistent with other studies (5, 47), our findings showed examples of athletes worrying about being viewed as undedicated to their sport if they were to become a mother. This might have hindered proactive discussions about pregnancy and mothering with coaches and club representatives. It remains unclear if there existed any mother athlete policies in the players’ clubs and the lack of communication seemed to be one explanation. Earlier and more transparent communication about pregnancy and maternity provisions has been recommended in several recent studies to improve the current situation in many sports (5, 6, 48). Recently, AC Milan was the first professional football club to guarantee automatic contract renewals for female players and staff in the event of pregnancy during the final season of their contracts (49). Further, in 2023, the PWHL included maternity leave in its collective bargaining agreement (50), which gives some reason to be optimistic about the future of women's ice hockey. However, adding automatic contract renewal for a sport that already faces economic challenges may be a challenging task for many sport clubs.

Traditionally, ice hockey has been a male dominated sport dominated by masculine norms. Further, until recently, women's ice hockey has been a low priority in many clubs (51). Hence, this could indicate that the sport is still in a transition phase between old and modern gender norms. Another explanation for the lack of clear maternity policies in the SDHL could be the relative rarity of mother-athletes within the league (e.g., no current mother-athletes). Possibly, this left the stakeholders unaware of the need for supporting athletes to initiate and maintaining the mother-athlete role. Examples of successful mother-athletes in other sports has shown that environmental support (e.g., from stakeholders) is essential for female athletes to initiate the mother-athlete role and to reach peak performance after childbirth (6, 43, 52). By contrast, lack of financial and social support during pregnancy and postpartum increases the risk for early career termination (5).

### 4.3 I can be that woman

The participants expressed several negative notions associated with the mother-athlete role. One possible explanation for this could be the lack of successful mother-athlete role models in the SDHL. Previous research illuminates that support from other mother-athletes has an important role in promoting knowledge and communication and help to optimize the mother-athlete support needed (5, 6). Unlike previous research findings (5, 51), the players did not express any concerns about being able to return physically to a comparable level of performance postpartum. Interestingly, this was despite the lack of current mother-athlete role models in the SDHL. One possible explanation could be that the money issue (e.g., low salaries) was too dominant to leave the players any room to even consider the physical impact of a pregnancy. Another explanation could be that there may be sport-specific differences (e.g., ice hockey and cross-country skiing). Taken together, our findings illuminate that there is a need for improving both financial support and for developing clear maternity policies for professional women's ice hockey in Sweden. This will enable more players to initiate the mother-athlete role and maintain a high level of performance postpartum. Further, current mother-athletes in the PWHL such as Kendall Coyne Schofield and Natalie Spooner will likely continue to challenge existing notions (e.g., that elite ice hockey and mothering are incompatible). Hopefully, this will also inspire more players to prolong their athletic careers as mothers. Since news media circulate gender ideologies and discourses as forms of truth, news media may have to be more aware of the how women's ice hockey is covered. For example, McGannon et al. (12) identified two mother-athlete identities represented in news media; (1) *athlete and mother in conflict* and (2) *athlete mother as superwoman.* Further, previous research show that such media representations may affect female athletes’ perceptions in multiple ways (e.g., notions about mothering) (12–14).

In the last decade, women's ice hockey has experienced several positive changes (e.g., increase in the number of players and several professional leagues) but there are still a lot to be done in terms of gender equality to make female players perceive the mother-athlete role as realistic. Future studies could also investigate how these challenges manifest for elite male athletes, in terms of a “father vs. athlete” conflict in professional ice hockey as well as explore the stakeholders’ (i.e., coaches, general managers, and representatives from the league) perspectives.

## 5 Conclusions

In this study we explored how Swedish elite female ice hockey players perceived challenges associated with initiating and maintaining the mother-athlete role. Our study offers valuable insights into female-specific challenges facing elite hockey players in Scandinavia considering motherhood. Thematic analysis (35, 36) revealed three main challenges in terms of: (a) *Making ends meet*, (b) *A leap in the dark*, and (c) *You can't be what you can't see.* These challenges made the players perceive the mother-athlete role as unrealistic. To change the current situation, our study identified continued work with improving the financial support and developing clear maternity policies as essential measures for stakeholders (e.g., sport federations, leagues and clubs) to take. This could prevent early retirement from elite sports in conjunction with having children. Further, enabling more elite players to prolong their athletic careers postpartum will likely help elite women's ice hockey to develop to its highest optimal quality (e.g., number of players and competition). Given our relatively small sample size, future studies could consider using quantitative approaches.

### 5.1 Limitations

The study design, using a qualitative approach with seven players could potentially “favor” the players’ perspective, because of their opportunity to elaborate and explain their own versions and interpretations of the situation. Accordingly, one limitation of the present study was that the stakeholders (i.e., coaches, general managers, and representatives from the league) were not interviewed. For example, the club might have had maternity rights that the players were unaware of. This could have generated a richer understanding of the environments that the participants were a part of. We authors acknowledge that the limited number of participants may be a limitation of the study. However, even if six general managers were contacted, only one of them replied to our request. Further, given that the present study showed examples of players who perceived even lifting the topic with their coaches and clubs as a potential risk of deselection, some players might have declined to participate based on that the request came from the coach/club and not directly from the researchers. Another explanation could be that the clubs didn't consider the research as relevant for them due to the absence of mother-athletes in the league. Additionally, the stressful life of many players (e.g., combining work and/or studies with the athletic career) may have left little time to participate in research projects. Despite this limitation with few participants, we argue that the dataset was sufficient in relation to the study aim with high “information power” (34) since the participants were “experts in their own experience” (51) as Swedish elite female ice hockey players considering motherhood.

## Data Availability

The raw data supporting the conclusions of this article will be made available by the authors, without undue reservation.

## References

[1] UN Women. Paris 2024 Olympics: A New Era for Women in Sport. New York, NY: UN Women (2024). Available online at: https://www.unwomen.org/en/paris-2024-olympics-new-era-for-women-in-sport

[2] International Olympic Committee. Women in the Olympic Movement. Lausanne, CH: International Olympic Committee (2024). Available online at: https://stillmed.olympics.com/media/Documents/OlympicMovement/Factsheets/Women-in-the-Olympic-Movement.pdf

[3] L’Heveder A Chan M Mitra A Kasaven L Saso S Prior T Sports obstetrics: implications of pregnancy in elite sportswomen, a narrative review. J Clin Med. (2022) 11(17):4977. 10.3390/jcm11174977 36078907 PMC9456821

[4] Solli GS Sandbakk Ø. Training characteristics during pregnancy and postpartum in the world’s most successful cross country skier. Front Physiol*.* (2018) 9:595. 10.3389/fphys.2018.00595 29875693 PMC5974210

[5] Bergström M Sæther SA Solli GS McGawley K. Tick-tock goes the biological clock: challenges facing elite Scandinavian mother-athletes. Women Sport Physic Act J. (2023) 32(1):1–9. 10.1123/wspaj.2022-0094

[6] Davenport MH Nesdoly A Ray L Thornton JS Khurana R McHugh TL. Pushing for change: a qualitative study of the experiences of elite athletes during pregnancy. Br J Sports Med. (2022) 56(8): 452–7. 10.1136/bjsports-2021-104755 35135828 PMC8995814

[7] McGregor B McGrath R Young J Nottle C. A scoping review of the experience of elite female athletes concerning pregnancy and motherhood. Sports Soc. (2023) 27(8):1221–53. 10.1080/17430437.2023.2270438

[8] Irani C Turner EHG Rumps MV Mulcahey MK. Recommendations for postpartum athletes returning to sport: the past, present, and future. Phys Sportmed*.* (2024) 52(6):533–40. 10.1080/00913847.2024.2385886 39082669

[9] Darroch F Schneeberg A Brodie R Ferraro ZM Wykes D Hira S Impact of pregnancy in 42 elite to world-class runners on training and performance outcomes. Med Sci Sports Exerc. (2023) 55(1):93–100. 10.1249/MSS.0000000000003025 35975937

[10] Bø K Artal R Barakat R Brown W Dooley M Evenson KR Exercise and pregnancy in recreational and elite athletes: 2016 evidence summary from the IOC expert group meeting, Lausanne. Part 2—the effect of exercise on the fetus, labour and birth. Br J Sports Med. (2016) 50(10):1297–305. 10.1136/bjsports-2016-096810 27733352

[11] Forstmann N Maignié A De Larochelambert Q Duncombe S Schaal K Maître C Does maternity during sports career jeopardize future athletic success in elite marathon runners? Eur J Sport Sci*.* (2022) 23(6):896–903. 10.1080/17461391.2022.2089054 35703008

[12] McGannon KR Gonsalves CA Schinke RJ Busanich R. Negotiating motherhood and athletic identity: a qualitative analysis of Olympic athlete mother representations in media narratives. Psychol Sport Exerc. (2015) 20:51–9. 10.1016/j.psychsport.2015.04.010

[13] McGannon KR Hladun W Kulkarni S Bundon A Pegoraro A. From birth to rebirth: comeback meanings in media stories of Canadian athlete mothers’ sporting journeys. Sport Educ Soc. (2024):1–15. 10.1080/13573322.2024.2376249

[14] McGannon KR McMahon J Gonsalves CA. Juggling motherhood and sport: a qualitative study of the negotiation of competitive recreational athlete mother identities. Psychol Sport Exerc. (2018) 36:41–9. 10.1016/j.psychsport.2018.01.008

[15] Tekavc J Wylleman P Ceci’c Erpič S. Becoming a mother-athlete: female athletes’ transitions to motherhood in Slovenia. Sport Soc. (2020) 23(4): 734–50. 10.1080/17430437.2020.1720200

[16] Appleby KM Fisher LA. Running in and out of motherhood: elite distance runners’ experiences of returning to competition after pregnancy. Women Sport Phys Act J. (2009) 18(1):3–17. 10.1123/wspaj.18.1.3

[17] Davenport MH Nesdoly A Ray L Khurana R Thornton JS McHugh TL. Is it realistic? A qualitative study of the experiences of elite women athletes considering parenthood. Sports Med*.* (2024) 54(9):2411–21. 10.1007/s40279-024-02019-y 38615294

[18] Massey K Whitehead AE. The myth that being a good mother and an elite athlete are not compatible. In: Coe J, editor. Book: Myths of Sports Performance. West Yorkshire: Sequoia Books (2024).

[19] Culvin A Bowes A. The incompatibility of motherhood and professional women’s football in England. Front Sports Act Living. (2021) 3:730151. 10.3389/fspor.2021.730151 34661099 PMC8514832

[20] McGregor B McGrath R Young J Nottle C. Exploring pregnancy and postpartum experiences among geographically diverse elite athletes: a qualitative study. J Sci Med Sport. (2024) 28(2):1–9. 10.1016/j.jsams.2024.10.001 39426848

[21] Pullen E Miller B Wiltshire G Plateau C. A feminist materialist inspired analysis of the meaning and management of pregnancy and reproductive health in Olympic and paralympic female athletes. Qual Res Sport Exerc Health*.* (2022) 15(3):332–44. 10.1080/2159676X.2022.2146162

[22] Martinez-Pascual B Alvarez-Harris S Fernández-De-Las-Peñas C Palacios-Ceña D. Maternity in Spanish elite sportswomen: a qualitative study. Women Health*.* (2014) 54:262–79. 10.1080/03630242.2014.883660 24512619

[23] International Ice Hockey Federation. Survey of Players. Zurich: International Ice Hockey (2023). Available online at: https://www.iihf.com/en/static/5324/survey-of-players

[24] Reid PA Mason DS. Women can’t skate that fast and shoot that hard!’ The first women’s world ice hockey championship, 1990. Int J Hist Sport. (2015) 32(14):1678–96. 10.1080/09523367.2015.1121867

[25] International Ice Hockey Federation. Women’s Ice Hockey. Zurich, CH. (2018). Available online at: https://www.iihf.com/en/static/5068/women-s-hockey

[26] Professional Women’s Hockey League. Collective Bargaining Agreement Between Professional Women’s Hockey League and Professional Women’s Hockey League Players Association. Toronto, CA: Collective Bargaining Agreement (2024). Available online at: https://www.thepwhl.com/en/faqs

[27] Svenska Damhockeyligan [Swedish Women’s Hockey League]. Den nya Spelaren [The New Player]. Stockholm: Svenska Damhockeyligan (2023). Available online at: https://www.sdhl.se/spelplanen

[28] Svenska Damhockeyligan [Swedish Women’s Hockey League]. (2024). Personal correspondence.

[29] Unionen. “Månadslönen för en Manlig Hockeyspelare—mer än vad ett Damlag Tjänar Sammanlagt” [“The Monthly Salary for One Male Hockey Player—More Than What a Women’s Team Earn Together”]. Stockholm: Unionen (2020). Available online at: https://www.unionen.se/pressmeddelande/3275168

[30] Stråhlman O. Elite Sport Career Process, Career Analysis of Former Swedish Elite Athletes. Göteborg: Gothenburg University Library (2006).

[31] Borchorst A Siim B. Woman-friendly policies and state feminism: theorizing Scandinavian gender equality. Fem Theory. (2008) 9(2):207–24. 10.1177/1464700108090411

[32] McKay AKA Stellingwerf T Smith ES Martin DT Mujika I Goosey-Tolfrey VL Defining training and performance caliber: a participant classification framework. Int J Sports Physiol Perform*.* (2022) 17:317–31. 10.1123/ijspp.2021-0451 34965513

[33] Archibald MM Ambagtsheer RC Casey MG Lawless M. Using zoom videoconferencing for qualitative data collection: perceptions and experiences of researchers and participants. Int J Qual Methods. (2019) 18:1–8. 10.1177/1609406919874596

[34] Malterud K Siersma VD Guassora AD. Sample size in qualitative interview studies: guided by information power. Qual Health Res*.* (2016) 26:1753–60. 10.1177/1049732315617444 26613970

[35] Braun V Clarke V. Thematic Analysis: A Practical Guide. Los Angeles, CA: SAGE (2022).

[36] Braun V Clarke V. Using thematic analysis in psychology. Qual Res Psychol*.* (2006) 3(2):77–101. 10.1191/1478088706qp063oa

[37] Sandelowski M. Whatever happened to qualitative description? Res Nurs Health. (2000) 22:334–40. 10.1002/1098-240x(200008)23:4<334::aid-nur9>3.0.co;2-g 10940958

[38] Tracy SJ. Qualitative quality: eight “big-tent” criteria for excellent qualitative research. Qual Inq. (2010) 16(10):837–51. 10.1177/1077800410383121

[39] Ponterotto JG. Qualitative research in counseling psychology: a primer on research paradigms and philosophy of science. J Couns Psychol. (2005) 52(2):126–36. 10.1037/0022-0167.52.2.126

[40] Baxter P Jack. Qualitative case study methodology: study design and implementation for novice researchers. Qual Rep. (2008) 13(4):544–59. 10.46743/2160-3715/2008.1573

[41] Colizza CR Bloom GA Loughead TM. Dual career in sport and non-sport work: exploring experiences of North American professional female ice hockey players. Psychol Sport Exerc. (2024) 74:102699. 10.1016/j.psychsport.2024.102699 38969305

[42] Hellborg AM. “Godispengar” Eller “Överdådig Lyx”: Om Elitidrott, Ekonomi och Jämställdhet [“Candy Money” or “Lavish Luxury”: On Elite Sports, Economics and Gender Equality]. Malmö: Malmö Studies in Sport Sciences (2019). Available online at: Idrottsforum.org

[43] Leavitt S Adams C. A successful failure? Troubling the road to the NWHL and professional women’s hockey. In: Bowes A Culvin A, editors. The Professionalization of Women’s Sports. Leeds: Emerald Publishing. (2019). p. 71–86. doi: 10.1108/978-1-80043-196-620211005

[44] Wilhelmsen L. Young and burned out—the dilemma of women’s elite football. Early termination of the football career for elite women footballers in Norway caused by a high degree of emotional and interpersonal stressors. Soccer Soc. (2023) 25(7):749–63. 10.1080/14660970.2023.2262925

[45] Statistikmyndigheten. Medellöner i Sverige [Average Salaries in Sweden]. Stockholm: Statistikmyndigheten (2024). Available online at: https://www.scb.se/hitta-statistik/sverige-i-siffror/utbildning-jobboch-pengar/medelloner-i-sverige/

[46] CBC. NCAA Star Daryl Watts Reveals Record $150,000 US Contract with PHF’s Toronto Six. Toronto, CA: CBC (2023). Available online at: https://www.cbc.ca/sports/hockey/phf-darylwatts-record-signing-1.6725545

[47] Culvin A. Football as work: the lived realities of professional women footballers in England. Manag Sport Leis. (2021) 28(6):684–97. 10.1080/23750472.2021.1959384

[48] McHugh T-L Davenport MH. Development of sport policy and practice recommendations for pregnant, postpartum and parenting Canadian high-performance athletes. BMJ Open Sport Exerc Med*.* (2024) 10(3):1–7. 10.1136/bmjsem-2024-001888 PMC11298730 39104377

[49] AC Milan. AC Milan Introduces Maternity Policy for Female Players and Staff. Milan: AC Milan (2024). Available online at: https://www.acmilan.com/en/news/articles/club/2024-08-02/ac-milan-introduces-maternity-policy-for-female-players-and-staff

[50] NHLPA. Opponents on the Ice, Athlete-Mothers Coyne Schofield and Spooner Share Common Bond. Toronto, CA: NHLPA (2024). Available online at: https://www.nhlpa.com/news/1-22673/opponents-on-the-ice-athlete-mothers-coyne-schofield-and-spooner-sharecommon-bond

[51] Stark T. Fullt Lag: Jämställdhet Inom Svensk Ishockey med Avseende på Kvinnors Inflytande Jämte Flick- och Damishockeyns Ställning [Full Team: Gender Equality Within Swedish Ice Hockey Focused on Women’s Influence on the Position of Girls’ and Women’s Ice Hockey]. Stockholm: Svenska Ishockeyföbundet (2022). Available online at: https://www.swehockey.se/media/p5/acunsr/sif-rapport-jamstalldhetdamishockey.pdf

[52] Dietz P Legat L Sattler MC van Poppel MNM. Triple careers of athletes: exploring the challenges of planning a pregnancy among female elite athletes using semi-structured interviews. BMC Pregnancy Childbirth. (2022) 22(1):643. 10.1186/s12884-022-04967-7 35971097 PMC9377111

